# Composition and Interactions of Hepatitis B Virus Quasispecies Defined the Virological Response During Telbivudine Therapy

**DOI:** 10.1038/srep17123

**Published:** 2015-11-24

**Authors:** Bin Zhou, Hui Dong, Yungang He, Jian Sun, Weirong Jin, Qing Xie, Rong Fan, Minxian Wang, Ran Li, Yangyi Chen, Shaoqing Xie, Yan Shen, Xin Huang, Shengyue Wang, Fengming Lu, Jidong Jia, Hui Zhuang, Stephen Locarnini, Guo-Ping Zhao, Li Jin, Jinlin Hou

**Affiliations:** 1 State Key Laboratory of Organ Failure Research, Guangdong Provincial Key Laboratory of Viral Hepatitis Research, Department of Infectious Diseases, Nanfang Hospital, Southern Medical University, Guangzhou, China; 2 Shanghai-MOST Key Laboratory of Health and Disease Genomics, Chinese National Human Genome Center at Shanghai, Shanghai, China; 3 CAS Key Laboratory of Computational Biology, CAS-MPG Partner Institute for Computational Biology; CAS Key Laboratory of Synthetic Biology, Institute of Plant Physiology and Ecology; Shanghai Institutes for Biological Sciences, Chinese Academy of Sciences, Shanghai, China; 4 Shanghai Shenyou Biotechnology Co., Ltd., Shanghai, China; 5 Department of Infectious Diseases, Ruijin Hospital, Shanghai Jiaotong University School of Medicine, Shanghai, China; 6 Department of Microbiology and Infectious Disease Center, School of Basic Medical Sciences, Peking University Health Science Center, Beijing, China; 7 Liver Research Center, Beijing Friendship Hospital, Capital Medical University, Beijing, China; 8 Victorian Infectious Diseases Reference Laboratory, North Melbourne, Victoria, Australia; 9 Department of Microbiology and Li Ka Shing Institute of Health Sciences, The Chinese University of Hong Kong, Prince of Wales Hospital, Shatin, New Territories, Hong Kong SAR, China; 10 State Key Laboratory of Genetic Engineering and Ministry of Education Key Laboratory of Contemporary Anthropology, School of Life Sciences and Institutes of Biomedical Sciences; Key Laboratory of Medical Molecular Virology affiliated to the Ministries of Education and Health, Shanghai Medical College and Department of Microbiology, School of Life Sciences; Fudan University, Shanghai, China; 11 Collaborative Innovation Center for Diagnosis and Treatment of Infectious Diseases, Zhejiang University, Hangzhou, China.

## Abstract

Reverse transcriptase (RT) mutations contribute to hepatitis B virus resistance during antiviral therapy with nucleos(t)ide analogs. However, the composition of the RT quasispecies and their interactions during antiviral treatment have not yet been thoroughly defined. In this report, 10 patients from each of 3 different virological response groups, i.e., complete virological response, partial virological response and virological breakthrough, were selected from a multicenter trial of Telbivudine treatment. Variations in the drug resistance-related critical RT regions in 107 serial serum samples from the 30 patients were examined by ultra-deep sequencing. A total of 496,577 sequence reads were obtained, with an average sequencing coverage of 4,641X per sample. The phylogenies of the quasispecies revealed the independent origins of two critical quasispecies, i.e., the rtA181T and rtM204I mutants. Data analyses and theoretical modeling showed a cooperative-competitive interplay among the quasispecies. In particular, rtM204I mutants compete against other quasispecies, which eventually leads to virological breakthrough. However, in the absence of rtM204I mutants, synergistic growth of the drug-resistant rtA181T mutants with the wild-type quasispecies could drive the composition of the viral population into a state of partial virological response. Furthermore, we demonstrated that the frequency of drug-resistant mutations in the early phase of treatment is important for predicting the virological response to antiviral therapy.

Globally, approximately one-third of the human population has been infected with the human hepatitis B virus (HBV), and 6% of infections develop into chronic infections^1^. To date, one of the effective medications for the treatment of human HBV infection is nucleos(t)ide analogs (NAs) that directly target the viral reverse transcriptase (RT), which is located at the polymerase gene^2^. Over the last two decades, several oral NA antiviral agents have been approved for chronic hepatitis B (CHB) treatment, with low or high genetic barriers against drug resistance. Although Entecavir (ETV) and Tenofovir Disoproxil Fumarate are both recommended as first-line therapies by major international guidelines^3^,^4^,^5^, these therapies have been difficult to implement for the majority of Asian-Pacific CHB patients due to economic concerns. Therefore, the low-genetic-barrier NAs are still extensively used, especially in countries where generic Lamivudine (LAM) and Adefovir Dipivoxil (ADV) are available, resulting in a high risk of partial virological response and drug resistance.

One possible reason for the development of NA resistance is that HBV quasispecies with drug-resistant mutations obtain a survival advantage under the selective pressure of antiviral drugs^6^,^7^. However, the process of drug resistance development is complex, and the interplay among various types of quasispecies may cause various levels of clinical virological responses. Biological studies of RNA viruses other than the DNA virus HBV suggest that internal cooperative or interfering interactions among quasispecies may determine the biological behavior of a viral population^8^,^9^,^10^. Mathematical models of competition-colonization dynamics have also been established for RNA viruses^10^,^11^. The relationship between the composition, frequency and interactions of HBV quasispecies and the later-phase virological responses in NA-treated patients has not yet been uncovered due to a lack of in-depth genetic analysis of systematically collected clinical samples. In this study, HBV quasispecies were examined with respect to critical drug resistance-related RT regions in time-serial samples from CHB patients who showed different virological responses to Telbivudine (LdT) therapy. Phylogenetic analyses and mathematical modeling were used to uncover the microevolution of wild-type and drug-resistant quasispecies and the dynamics of their relationship.

## Results

### Patient characteristics

The baseline characteristics of the 30 enrolled patients were as follows: median age was 31.5 years; 24 (80%) patients were male; median baseline levels of HBV DNA and HBsAg were 9.09 Log_10_ copies/mL and 4.60 Log_10_ IU/mL, respectively; and median baseline ALT levels were 130.5 IU/L. Table 1 and Supplementary Table S1 present the clinical characteristics and the serum HBV DNA levels of the enrolled patients.

### Different distributions of drug-resistant mutations among patient groups

The aforementioned 107 samples were subjected to ultra-deep sequencing using the 454 GS FLX+ System. The sequenced DNA segments covered the critical LdT-resistance region of the RT gene^2^,^5^. A total of 496,577 sequence reads were obtained. The average sequencing coverage was 4,641X per sample. To avoid any possible false-positive results caused by low sequencing quality, only the nucleotide reads with a quality score ≥20 were included in the following analyses. The 454 ultra-deep sequencing results of the time-serial samples from each subject were compared with their corresponding references, i.e., the Sanger sequencing data of the week-0 samples. Any mismatches between the 454 GS FLX+ sequencing results and the reference sequence were considered mutations. The prevalence of mutations among all of the samples is shown in Supplementary Table S4.

**Figure 1:**
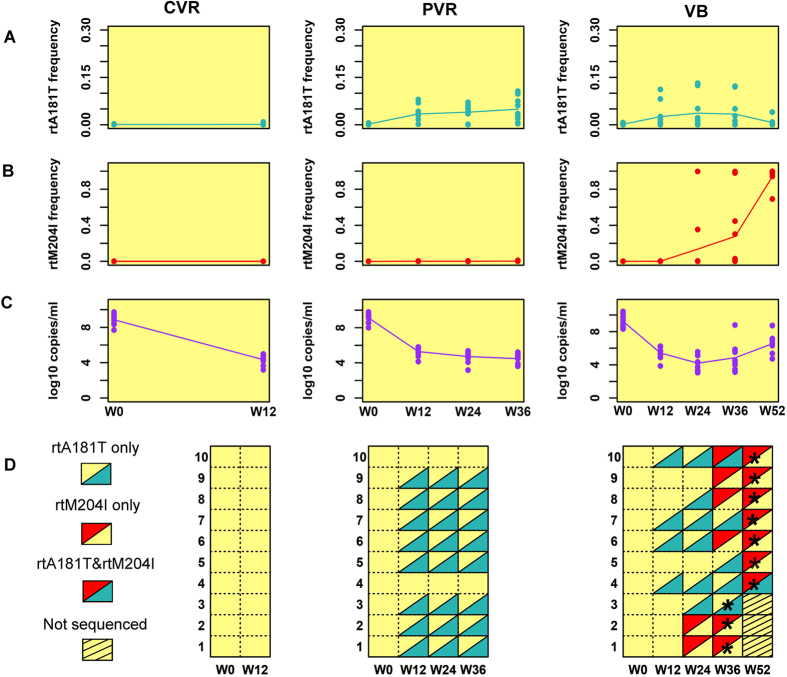
Dynamics of the rtA181T and rtM204I mutants and patient HBV DNA levels in the CVR (left panel), PVR (central panel) and VB (right panel) groups. In each panel, four separate figures independently present the frequencies of rtA181T or rtM204I mutants (**A,B**) respectively), the HBV DNA levels (**C**), and a visualization of the presence or absence of the critical mutants (**D**). The colored dots show values for individual observations at weeks 0, 12, 24, 36 or 52, whereas the corresponding lines show their average trends. A black star indicates the time point when the virological breakthrough occurred. The left panel shows that both the rtA181T and rtM204I mutants were absent from the CVR group. The central panel shows that the rtM204I mutant was also absent from the PVR group but that the rtA181T mutant was continuously observed in 8 out of 10 subjects from week 12 to week 36. The right panel shows that the rtA181T mutant was detected in 7 of 10 subjects in the early stage of the treatment but that its frequency decreased after the virological breakthrough (week 36 or 52). Meanwhile, the rtM204I mutant was detected in 9 out of 10 VB subjects.

**Figure 2:**
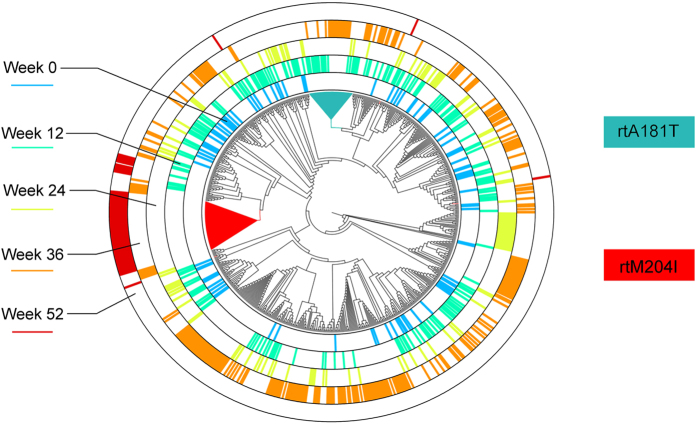
Phylogenies of the HBV strains from patient VB.7 with a typical virological breakthrough. Patient VB.7 experienced a virological breakthrough at week 52 of the LdT treatment. The rtA181T mutant was detectable at weeks 12, 24 and 36, whereas the rtM204I mutant was detectable at week 52. The lineages carrying rtA181T and rtM204I are marked in blue and red, respectively. This coloring indicates that these two mutants were derived from independent phylogenetic origins. The pairwise difference of the subject’s viral sequences at each time point are shown on different circular rims in different colors (week 0, sky blue; week 12, light green; week 24, yellow green; week 36, orange; week 52, dark red). A significant decrease in the pairwise difference was observed at week 52 (after the virological breakthrough) compared with week 36 (before the virological breakthrough).

Two critical LdT-resistant mutations, rtA181T and rtM204I, were distributed differently in the three patient groups (Fig. 1, Supplementary Table S2). These two mutants were derived from independent phylogenetic origins and therefore should be considered as two separate quasispecies (Fig. 2, Supplementary Figure S1).

In the complete-virological-response (CVR) group (Fig. 1A, Supplementary Table S2), neither of the critical mutations was detected with a frequency ≥1%. In the partial-virological-response (PVR) group (Fig. 1B, Supplementary Table S2),, the rtA181T mutant was not detected with a frequency ≥1% in any of the subjects at baseline (week 0), but it was continuously found from week 12 to week 36 in 8 (patients PVR. 1–3 and 5–9) of the 10 subjects, with frequencies varying from 1.7% to 10.6%. The rtM204I mutant was not detected at a frequency ≥1% in any of the examined samples from the PVR group. Because the rtA181T substitution at the RT region plays an essential role in development of resistance to treatments with LdT 7,12, this novel observation regarding its quantitative dynamic variation during the course of drug treatment in the PVR group suggested that rtA181T mutants might be responsible for the patients’ partial virological responses to LdT. In the virological-breakthrough (VB) group (Fig. 1C, Supplementary Table S2), neither rtA181T nor rtM204I was found at a frequency ≥1% in any of the subjects at baseline measurement. When the VB occurred, either at week 36 (in patients VB.1-3) or week 52 (in patients VB.4–10), the rtM204I mutant was detected in 9 (patients VB.1–2 and 4–10) of 10 subjects, and it accounted for a dominant portion of the viral population in these subjects. Notably, among these 9 patients, the rtA181T mutant was detectable in six of them (patients VB.4–8 and 10) either 24 (patients VB.6, 10) or 40 (patients VB.4, 5, 7) weeks earlier than when rtM204I was detected; however, its frequency dropped below 1% once the frequency of rtM204I increased to 90% (Fig. 1D, Supplementary Table S2). Furthermore, the rtM204I mutant could be detected 12 weeks (patients VB.1, 2) or 16 weeks (patients VB.6, 8–10) earlier than the VB. Although the rtM204I mutant was not detected at any of the time points from baseline to VB in patient VB.3, the rtA181T mutant was detected 12 weeks earlier than when its VB occurred (Fig. 1D, Supplementary Table S2).

### Cooperation between the rtA181T mutant and wild-type viruses

The rtA181T mutation also causes a stop-codon mutation in the overlapping surface protein (S protein) gene (Supplementary Table S3), i.e., sW172*, which causes a 55 amino acid-residue truncation in the S protein. Hence, the rtA181T/sW172* mutants need the aid of coexisting wild-type quasispecies to provide a functional S protein for virion packaging^13^,^14^. To understand the mechanism of co-existence between the rtA181T mutant and wild-type viruses in PVR patients, we theoretically modeled their cooperative relationship, highlighting the complementary nature of their RT and S proteins.

We established a mathematical model of HBV dynamics (Supplementary Text S1-S3), in which the differences in viral replication ([*β*_*U*_*P*_*U*_ + *β*_*M*_(1- *P*_*U*_)][*δP*_*U*_ + (1- *P*_*U*_)]*L*) and viral clearance (*γL*) at a given time would determine the changing rate of the viral load (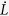) over the specified time. Viral replication was regulated by polymerase activities (*β*_*U*_ and *β*_*M*_), the frequency of the specified mutants in the population (*P*_*U*_), the relative packaging efficiency of the S protein (*δ*) of the specified mutants, and the viral load at the moment (*L*). The viral clearance is determined by both the clearance efficiency (*γ*) and the viral load at the moment (*L*).

A theoretical analysis based on the above simulation showed that relatively low packaging efficiency (*δ *≤ 0.5) prevents rtA181T mutants from achieving dominance when coexisting with the wild-type quasispecies, in accord with the observed frequencies of rtA181T mutants (1.7 ~ 10.6%) in the viral populations of PVR patients (Supplementary Table S2). By contrast, when drug-resistant mutants such as rtM204I, which causes a function-preserving (*δ* ≈ 1) sW196L mutation in the overlapping S protein (Supplementary Table S3), coexist with wild-type viruses in a population, the model predicts that those mutants can dominate the population. This prediction is consistent with the observation that the frequency of drug-resistant rtM204I mutants reached nearly 100% in VB patients (Supplementary Table S2).

### Competition between rtM204I mutants and other quasispecies

**Figure 3:**
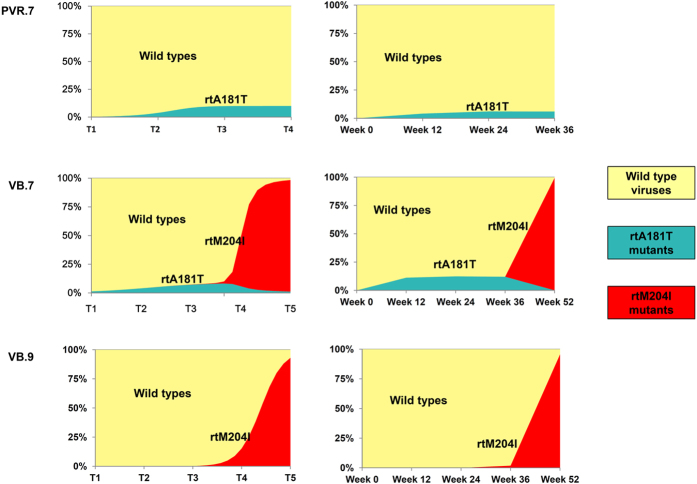
Dynamics of the wild-type and mutated HBV strains in the computer simulation. The dynamics of the frequencies of wild-type HBV and the rtA181T and rtM204I mutants during LdT treatment are marked in yellow, blue and red, respectively. The left panel represents the result of the computer simulations of three representative subjects (VB.7 (**A**), VB.9 (**B**) and PVR.7(**C**)), whereas the right panel shows the results obtained from 454 sequencing. In all three subjects, the left panel matches the right panel quite well, indicating the computer simulations could reproduce the experimental sequencing observations.

To further inspect the competition between rtM204I and other quasispecies, we simulated the dynamics of the quasispecies during antiviral treatment (Fig. 3 and Supplementary Text S1-S4). In a simulation with the presence of rtM204I mutants, the mutants will eventually dominate the entire population. When the proportion of rtA181T mutants is much greater than that of rtM204I mutants at baseline, the proportion of rtA181T mutants could rise to approximately 10% of the total viral population before decreasing rapidly as the proportion of rtM204I mutants increased (Fig. 3A). In contrast to the abundance of rtM204I mutants, the rtA181T mutants never reach a significant level if present at a proportion similar to or lower than that of the rtM204I mutants at baseline (Fig. 3B). In a viral population without an rtM204I mutant, the frequency of rtA181T mutants increases to approximately 10% and then stabilizes (Fig. 3C). The simulated dynamic patterns coincided well with the corresponding clinical observations, which indicated that rtM204I mutants have a significant advantage in competitive survival, even in the presence of drug-resistant rtA181T mutants.

### Compositions and frequencies of the quasispecies associated with virological responses

To investigate the relationships between the compositions and frequencies of the drug-resistant mutants at the early phase and the virological responses to treatment, we examined the time-serial data from all three patient groups in their early-phase samples. The frequencies of rtA181T mutants were significantly lower in the CVR patients than in the other two sub-optimal groups at baseline (*p* ≤* *0.023 in the permutation test^15^). Furthermore, at week 12 and thereafter, the frequencies of rtA181T mutants were higher than 1% in 15 of the 20 PVR and VB subjects but in none of the 10 CVR patients (Supplementary Table S2). Therefore, the proportion of the rtA181T mutant in the patient at the early phase of treatment (including baseline) can distinguish patients who will experience the optimal response (i.e., CVR) from those who will experience sub-optimal responses (i.e., PVR and VB). At baseline and week 12, the rtM204I mutants were observed to be absent or extremely rare. However, at week 24 and thereafter, the frequency of rtM204I mutants reached 1% or greater in 9 of the 10 VB patients but remained at zero or less than 1% in the PVR patients (*p* ≤ 1.19E-4 in Fisher’s exact test)^16^. Therefore, the proportions of rtA181T and rtM204I mutants at the early stage of treatment were significantly different among the CVR, PVR and VB groups, thus implying a potential association between quasispecies composition and later-phase virological responses.

## Discussion

In this study, HBV quasispecies were profiled in a time series of samples from LdT-treated CHB patients who showed different responses to antiviral therapy. By combining ultra-deep sequencing with phylogenetic analyses and mathematical modeling, we investigated the dynamics of drug-resistant and other quasispecies in the HBV population and revealed the cooperation-competition relationship among these quasispecies. Our results also suggest that quasispecies composition at the early stage of treatment may be associated with later-phase virological responses.

Constellations of mutations required for adaptability may be located either in the same genomic molecule or in a number of different genomes that may complement each other^17^,^18^. Although we could not completely rule out the possibility that rtA181T and rtM204I simultaneously exist in the same genome due to the limited sequencing coverage in this study, our results indicated that most of the rtA181T and rtM204I mutants were derived from independent phylogenetic origins and therefore should be considered as two separate quasispecies. The independent origins of these two well-characterized drug-resistant mutants suggest that rtA181T and rtM204I double mutants may not possess a significantly greater evolutionary advantage than those carrying a single mutation. Meanwhile, the rtA181T/sW172* mutants achieved evolutionary superiority by being functionally complementary with other quasispecies carrying the wild-type S gene (the quasispecies mutually share functional RT enzymes and S proteins)^13^. Quasispecies with a functional drug-resistant RT enzyme and a defective S protein, such as rtA181T/sW172* mutants, may survive without requiring rescue from the defective S protein by additional mutations^19^. This functional complementation not only results in the maintenance of a low but relatively constant viral population, as seen in the PVR status under LdT treatment, but it also allows the eventual development of more-effective drug-resistance mutants over the course of antiviral therapy. In this regard, the rtM204I mutants, which carry both a drug-resistant RT enzyme and a functional S protein, clearly have superior fitness than either rtA181T/sW172* mutants or wild-type viruses in the presence of LdT. Although packaging-deficient rtA181T/sW172* mutant can dominate in viral populations during LAM or ADV treatment^20^, our results showed that it always accounted for a minor proportion of the viral population during LdT treatment, thus suggesting that the composition of viral quasispecies may vary under the different selection pressures of various drugs.

Suboptimal responses (PVR and VB) after antiviral treatment, as defined in the EASL guidelines^3^, are prevalent not only in patients treated with low- genetic-barrier drugs but also in those treated with high-genetic-barrier drugs^3^,^21^. The mechanisms of the suboptimal responses have not been fully elucidated and may be related to the potency of drugs^21^, poor patient adherence^22^, drug metabolism^21^, or quasispecies/mutations of HBV genes^23^. One pioneering study using Sanger sequencing technology investigated the microevolution of the RT region within HBV quasispecies during the early stage of ETV and LAM treatment^23^. It was found that the genetic complexity of the viral population at week 4 was significantly lower in responders than in partial responders. A preliminary theory stated that various quasispecies evolved along the course of treatment, and an attempt at applying this theory sought to differentiate responders from partial responders as early as week 4^23^. In the present study, with the aid of ultra-deep sequencing technology, we were able to examine the microevolution of large populations of RT-related drug-resistance mutations in time-series samples, albeit within a limited region of a single target gene. Our results showed that the rtA181T/sW172* mutant was present at time points after week 12 in most patients who showed a suboptimal response to LdT. This result stood in contrast to the absence of this mutant in all of the patients who showed a CVR. Because the rtA181T/sW172* mutant shows resistance to LdT^5^,^7^, it is likely that rtA181T and wild-type viruses represented a complimentary pair of quasispecies that gained a certain extent of viral fitness and whose presence manifested as partial resistance to LdT. This partial resistance resulted in the finding that LdT could not suppress the virus to the necessary low levels. Therefore, it is reasonable to suggest that drug-resistance mutations play an important role not only in the development of the VB but also in the development of the PVR.

The clinical relevance of suboptimal virological responses relates to a high risk of developing resistance to long-term anti-HBV treatment^21^. This study actually substantiates this scenario with exact data about quasispecies evolution. Among the 15 patients in this study who showed suboptimal virological responses and exhibited the rtA181T/sW172* quasispecies at week 12 or beyond, 7 of them eventually progressed to VB (Fig. 1D), supporting the argument that synergistic growth of rtA181T/sW172* with the wild-type quasispecies may facilitate the development of VB. Additionally, once the rtM204I mutant prevailed over the rtA181T and wild-type viruses, we observed that VB developed quickly and that rtM204I predominated among the viral quasispecies, supporting the argument that the rtM204I mutant may have greater fitness than the rtA181T mutant under LdT treatment. These results indicate that the response to antiviral treatment might be predicated upon the composition and frequency of drug-resistant quasispecies that are present at the early stage of treatment.

However, it is controversial whether drug-resistant mutants originate from quasispecies at the baseline or develop during antiviral treatment^24^,^25^,^26^. Because the HBV viral load decreased dramatically under antiviral therapy (Fig. 1), it is unlikely that the drug-resistance mutations arose in the viral population during treatment. We calculated the accumulation rates of mutation events before and after antiviral treatment (Supplementary Text S5). Assuming a constant mutation rate and an exponential change of viral load, the rate of accumulation of mutational events decreases rapidly after the beginning of treatment. For instance, the number of mutation events occurring in the 6th week might be only 1.5%(0.015) that in the 1st week. Furthermore, the number of mutational events occurring over a one-year treatment period (52 weeks) might be only 1/3 (0.325) that in the month immediately preceding the treatment. Because the number of mutational events occurring during the treatment is much lower than before treatment, most of the mutants may have existed in the HBV quasispecies pool before treatment, although their frequencies may be too low to be reliably detected. In our study, both the rtM204I and rtA181T mutants were found at low frequencies (<1%) in some of the CVR, PVR and VB subjects at baseline (Supplementary Table S2).

There are several limitations to this study. First, although deep sequencing is informative, bias resulting from PCR and relatively high error rates introduced by the sequencing system are inevitable. To avoid false-negative calls in cases of extremely low-frequency variants and false-positive calls in the detected variants, several experimental control and calculation steps were taken in this study. We used a conservative cutoff of 1% to distinguish authentic mutations from sequencing errors; this cutoff may have also led to missing information about the rtA181T and rtM204I mutations if present at proportions less than 1%. Second, the present findings were only observed in a LdT-treated cohort. Further research using this approach must be conducted in patients treated with other NAs to substantiate the results. Despite these limitations, our study demonstrated for the first time that the dynamics of different viral quasispecies in the RT region are associated with distinct virological response outcomes.

In conclusion, our in-depth analysis of rtA181T and rtM204I mutants suggests that patient virological responses to antiviral therapy are determined by the presence and composition of critical drug-resistant mutants at an early stage of treatment. This investigation identified details about the mechanism of drug-resistance selection in this clinical cohort and provided the opportunity to perform sophisticated molecular phylogenetic analysis, which in turn permitted the development of a mathematical model that successfully led to an in-depth understanding of microevolution in a viral quasispecies population. Further technological improvements for accessing more accurate sequence reads with lower viral loads and other research advances using approaches similar to those of this study may eventually lead to the application of such techniques for personalized antiviral therapy.

## Patients and Methods

### Patients and laboratory evaluation

**Figure 4:**
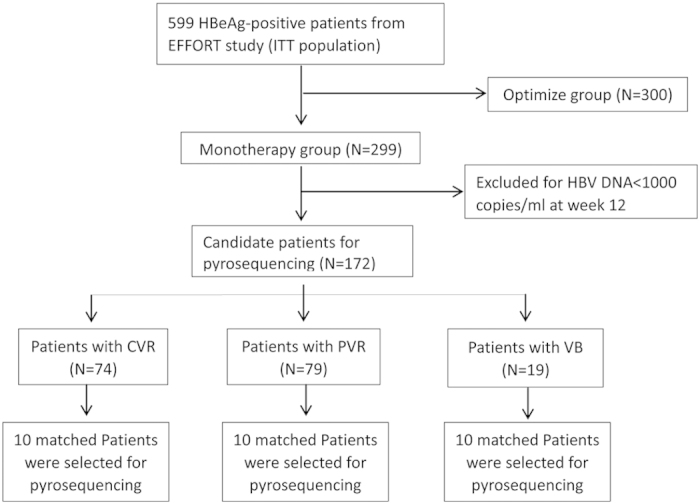
A flowchart of the patients enrolled in the study. Patients in the OPTIMIZE group (Group I) started LdT 600 mg daily from the baseline, and 10 mg of daily ADV was added to patients with suboptimal responses from week 28. Patients in the MONO group (Group II) started LdT monotherapy from the baseline. All of the patients with LdT monotherapy had ADV added once a confirmed virological breakthrough had developed. ITT, intention to treat.

The patients analyzed in our study were selected from a phase-IV, 2-year, multicenter, randomized, controlled trial with LdT ± ADV treatment (EFFORT study, NCT00962533). The detailed study design and the main efficacy results of this trial have been published^27^,^28^. In this trial, 599 NA-naive CHB patients were randomly allocated to 2 groups with different treatment strategies, namely Group I (300 patients) and Group II (299 patients). Only the LdT monotherapy group (group II) was involved in our study. To achieve accurate monitoring of the dynamics of HBV quasispecies by ultra-deep pyrosequencing, 127 patients with HBV DNA <1,000 copies/ml (171.82 IU/ml) at week 12 were not considered for further genetic studies because their serum samples did not provide enough HBV DNA for sequencing. The remaining 172 patients were divided into three groups according to their virological responses to LdT monotherapy: the CVR (complete virological response) group, whose serum HBV DNA was ≥1,000 copies/ml (171.82 IU/ml) at week 12 and persistently <1,000 copies/ml (171.82 IU/ml) from week 24 to week 52 of the treatment and who did not have a virological breakthrough; the PVR (partial virological response) group, whose serum HBV DNA was persistently ≥1,000 copies/ml (171.82 IU/ml) from week 12 to week 52 of the treatment and who did not have a virological breakthrough; and the VB (virological breakthrough) group, whose serum HBV DNA was increased by ≥1 log_10_ above its nadir during week 12 to the week-52 treatment. Ten subjects with matched HBV DNA levels, genotypes, and HBsAg levels at baseline were selected from each of the three patient groups (CVR group 10/74, PVR group 10/79, and VB group 10/19). In total, 107 samples (20 samples, from week 0 and week 12 for each patient in the CVR group; 40 samples, from weeks 0, 12, 24 and 36 for each patient in the PVR group; and 47 samples, from weeks 0, 12, 24, 36 and/or 52 for patients in the VB group) were subjected to ultra-deep sequencing using a 454 GS FLX+ System. A flowchart of the patients enrolled in this study is shown in Fig. 4.

The study was approved by the Ethics Committee of Nanfang Hospital, Southern Medical University. Written informed consent was obtained from all of the patients. All of the experiments were performed in accordance with approved guidelines of Southern Medical University.

Laboratory assessments were conducted every 12 weeks from baseline to the end of the study. HBV DNA levels and HBV serological markers were measured with the Roche COBAS TaqMan platform (with a lower limit of detection of 12 IU/mL or 69.84 copies/mL) and with an ARCHITECT i2000SR in the central laboratory, respectively. Serum ALT levels were assessed at local laboratories according to standard procedures. Genotypes were determined by sequencing the small S region and by using the phylogenetic method described previously^29^.

### HBV DNA extraction and deep sequencing

HBV DNA was extracted from the sera of the 107 patients using a QIAamp UltraSens Virus kit (Qiagen, Hilden, Germany) according to the manufacturer’s protocol. A pair of fusion primers was designed to amplify a fragment of the HBV reverse transcriptase (RT) gene spanning from amino acid 96 to 217, a region within which the mutations known to be responsible for LdT resistance are located. The fusion primers consisted of 454 GS adaptor sequences at the 5′ end, followed by a sample-specific barcode and HBV-specific primers at the 3′ end. The sequences of the forward (F) and reverse (R) primers were 5′-CCATCTCATCCCTGCGTGTCTCCGACTCAGXXXXXXXXGCTGCTATGCCTCATCTTC-3′ (415–433 nt), and 5′-CCTATCCCCTGTGTGCCTTGGCAGTCTCAGXXXXXXXXTCAAGATGYTGTACAGACTT-3′ (782–763 nt), respectively (the string of “X” characters indicates the sample-specific barcode sequence). The PCR reaction was performed in a total volume of 50 μl that contained 1 μl of extracted HBV DNA, 1× Buffer, 0.2 mM dNTPs, 1.0 mM MgSO_4_, 0.3 μM fusion primers F/R, and 1.0 unit of KOD Plus DNA polymerase (TOYOBO, Osaka, Japan). The PCR products were purified using a QIAQuick PCR Purification kit (Qiagen, Hilden, Germany) and were mixed together at an equimolar concentration for amplification by emulsion PCR. Ultra-deep sequencing was then performed on a 454 GS FLX+ System (Roche, Basel, Switzerland). The distribution of the sequence read length is shown in Supplementary Figure S2. The read length of most sequences converged at approximately 380 bp, matching exactly the length of the PCR products that were sequenced.

It was suggested that internal controls tailored to the amplicon fragment of interest should be included in the 454 sequencing due to its highly non-random nature of errors^30^,^31^. Thus, we employed an amplicon obtained from a plasmid as an experimental control for estimating the sequencing error rate. A plasmid containing the HBV RT region was cloned from a treatment-naïve patient and was used as the template for PCR amplification with the same primers listed above. The amplicon was then sequenced in parallel by the 454 GS FLX^+^ System, and the Sanger method was used to evaluate the sequencing errors of the 454 GS FLX^+^ System. Taking the Sanger sequencing result of the plasmid as a reference sequence, any mismatches between the sequencing result of the 454 GS FLX^+^ System and the Sanger method were considered sequencing errors. The plasmid was sequenced together with the clinical samples in 6 separate runs and showed an overall mismatch error rate of 0.016%. Over the 6 sequencing runs, a mean of 1.3 nucleotides per sequence was observed to have a mismatch error rate ≥1% (Supplementary Figure S3). Thus, we took a conservative cutoff of 1% to distinguish authentic mutations from sequencing errors, a standard that was adopted in previous studies on ultra-deep sequencing of HBV^31^,^32^.

The Sanger sequencing results of week-0 samples from each patient were used as reference sequences for the analysis of their 454 sequencing data obtained from each time point. Mismatched bases between the 454 sequencing data and the reference sequences were regarded as mutations. A reliably identified mutation was expected to meet the following criteria: 1) frequency of mutation ≥1%; 2) quality score ≥20; and 3) coverage ≥500.

### Reconstructing phylogenetic trees of patient HBV sequences

All of the sequence reads from a single patient were aligned using the MUSCLE (version 3.8.31) software with default parameters^33^. To achieve a clear presentation, we applied a multi-step procedure to remove redundant sequences from the initial sequence set. In the first step, we sequentially removed sequences with high similarity to any others until the p-distance of the remaining sequences was ≥0.5%. Next, the remaining sequences were aligned again using the same software and default parameters. A phylogenetic tree of the remaining sequences for each subject was reconstructed using the neighbor-joining (NJ) algorithm, and a maximum composite likelihood substitution model was implemented in MEGA5. The reconstructed NJ trees and collection dates of the samples were visualized using FigTree^34^ (version 1.3.1) and MATLAB (version 7.13).

### Dynamic modeling

A system of ordinary differential equations was implemented for modeling the dynamics of quasispecies. We separately specified the total amounts of rtA181T, rtM204I mutants, and drug-sensitive wild-type viruses in the system. The rate of fluctuation in the total viral amount is determined by the difference between the rates of viral replication and clearance. Dynamic-model HBV dynamics were modeled according to the methods of Nowak MARM^35^ (details are presented in Supplementary Text S1-S3).

### Computer simulation

The observed change of rtA181T and rtM204I mutant frequencies in the PVR and VB groups enabled us to assign parameters for the aforementioned dynamic model and further explore the mechanism of HBV dynamics during antiviral therapy (details are presented in Supplementary Text S4). In cases with rtA181T mutants, the decreased packaging efficiency of the deficient S protein was coupled with the enhanced activity of the mutated RT enzyme under NA treatment^13^.

## Additional Information

**How to cite this article**: Zhou, B. *et al.* Composition and Interactions of Hepatitis B Virus Quasispecies Defined the Virological Response During Telbivudine Therapy. *Sci. Rep.* **5**, 17123; doi: 10.1038/srep17123 (2015).

## Supplementary Material

Supplementary Materials

Supplementary Table S4

## Figures and Tables

**Table 1:** Baseline clinical characteristics of patients enrolled in this study.

Groups	CVR (N = 10)	VB (N = 10)	PVR (N = 10)	P-value
Male	8	7	9	0.535
Age, years	31.2 ± 11.2	37.3 ± 11.3	29.3 ± 7.8	0.206
HBV sub-genotype				0.287
B2	6	3	3	
C2	4	7	7	
Baseline HBV DNA levels, Log_10_ copies/mL				
Median (range)	8.95 (7.68–9.72)	9.19 (8.31–10.42)	9.26 (7.99–9.78)	0.371
Mean ± SD	8.86 ± 0.62	9.23 ± 0.75	9.11 ± 0.58	0.431
Baseline HBsAg levels, Log_10_ IU/mL				
Median (range)	4.45 (3.18–4.98)	4.70 (3.71–5.46)	4.54 (3.35–4.74)	0.939
Mean ± SD	4.34 ± 0.56	4.66 ± 0.63	4.35 ± 0.44	0.347
Baseline ALT Levels, IU/mL				
Median (range)	150.65 (91–676)	116.45 (45–193)	126.95 (82–622)	0.196

Notes: HBV DNA levels were determined using Roche COBAS TaqMan. HBsAg levels were assayed using ARCHITECT i2000SR. CVR: complete virological response group; PVR: partial virological response group; VB: virological breakthrough group; ALT: alanine aminotransferase; ULN, upper limit of normal; and HBsAg, hepatitis B surface antigen.
