# Supplementary material for: Composition and Interactions of Hepatitis B Virus Quasispecies Defined the Virological Response During Telbivudine Therapy
Source: Sci Rep. 2015 Nov 24;5:17123. doi: 10.1038/srep17123 (PMC4657086; doi:10.1038/srep17123)
Supplement: Supplementary Materials [file srep17123-s1.doc]

**Composition and Interactions of Hepatitis B Virus Quasispecies Defined the Virological Response during TELBIVUDINE Therapy**

**Authors:**

Bin Zhou^1†^, Hui Dong^2†^, Yungang He^3†^, Jian Sun^1†^, Weirong Jin^2,4†^, Qing Xie^5^, Rong Fan^1^, Minxian Wang^3^, Ran Li^3^, Yangyi Chen^2^, Shaoqing Xie^4^, Yan Shen^4^, Xin Huang^3^, Shengyue Wang^2^, Fengming Lu^6^, Jidong Jia^7^, Hui Zhuang^6^, Stephen Locarnini^8^, Guo-Ping Zhao^2,3,9,10*^, Li Jin^3,10*^, Jinlin Hou^1,11*^

**Affiliations:**

^1^. State Key Laboratory of Organ Failure Research, Guangdong Provincial Key Laboratory of Viral Hepatitis Research, Department of Infectious Diseases, Nanfang Hospital, Southern Medical University, Guangzhou, China

^2^. Shanghai-MOST Key Laboratory of Health and Disease Genomics, Chinese National Human Genome Center at Shanghai, Shanghai, China

^3^. CAS Key Laboratory of Computational Biology, CAS-MPG Partner Institute for Computational Biology; CAS Key Laboratory of Synthetic Biology, Institute of Plant Physiology and Ecology; Shanghai Institutes for Biological Sciences, Chinese Academy of Sciences, Shanghai, China

^4^. Shanghai Shenyou Biotechnology Co., Ltd., Shanghai, China

^5^. Department of Infectious Diseases, Ruijin Hospital, Shanghai Jiaotong University School of Medicine, Shanghai, China

^6^. Department of Microbiology and Infectious Disease Center, School of Basic Medical Sciences, Peking University Health Science Center, Beijing, China

^7^. Liver Research Center, Beijing Friendship Hospital, Capital Medical University, Beijing, China

^8^. Victorian Infectious Diseases Reference Laboratory, North Melbourne, Victoria, Australia.

^9^. Department of Microbiology and Li Ka Shing Institute of Health Sciences, The Chinese University of Hong Kong, Prince of Wales Hospital, Shatin, New Territories, Hong Kong SAR, China

^10^. State Key Laboratory of Genetic Engineering and Ministry of Education Key Laboratory of Contemporary Anthropology, School of Life Sciences and Institutes of Biomedical Sciences; Key Laboratory of Medical Molecular Virology affiliated to the Ministries of Education and Health, Shanghai Medical College and Department of Microbiology, School of Life Sciences; Fudan University, Shanghai, China

^11^Collaborative Innovation Center for Diagnosis and Treatment of Infectious Diseases, Zhejiang University, Hangzhou

^†^ These authors contributed equally to this work.

**Corresponding authors:**

Jinlin Hou (jlhousmu@163.com); Li Jin (lijin@fudan.edu.cn) or Guo-Ping Zhao (gpzhao@sibs.ac.cn);

**Supplementary Materials**

**Supplementary text S1: Dynamic modeling.**

A system of ordinary differential equations was implemented for modeling dynamics of quasispecies during the therapy. We presented the total amounts of rtA181T, rtM204I mutants, and drug-sensitive virus in the system as LA, LB and LC, respectively. Rate of fluctuation of the total viral amount is determined by the difference between rates of viral replication and clearance. In our model, the rates of viral replication are affected by activities of polymerase (noted by βA, βB, and βC), relative affinity of polymerases (noted by αA, αB, and αC,) and viral amounts at the moment (LA, LB, and LC). Further, we assumed that the rates of viral clearance are determined by a constant clearance efficiency γ and corresponding viral amounts at the moment (LA, LB, and LC). The system of ordinary differential equations (Eq. 1-3) is presented below:

$$\begin{matrix} \frac{dL_{A}}{dt}=(\beta_{A}\frac{\alpha_{A}L_{A}}{\alpha_{A}L_{A}+(1-\alpha_{A})(L_{B}+L_{C})}+\beta_{B}\frac{(1-\alpha_{A})L_{B}}{\alpha_{A}L_{A}+(1-\alpha_{A})(L_{B}+L_{C})}+\beta_{C}\frac{(1-\alpha_{A})L_{C}}{\alpha_{A}L_{A}+(1-\alpha_{A})(L_{B}+L_{C})}) \\ \times(\frac{\delta\alpha_{A}L_{A}}{\alpha_{A}L_{A}+(1-\alpha_{A})(L_{B}+L_{C})}+\frac{(1-\alpha_{A})(L_{B}+L_{C})}{\alpha_{A}L_{A}+(1-\alpha_{A})(L_{B}+L_{C})})L_{A}-\gamma L_{A}\text{ (Eq.1)} \end{matrix}$$

$$\begin{matrix} \frac{dL_{B}}{dt}=(\beta_{A}\frac{(1-\alpha_{B})L_{A}}{(1-\alpha_{B})(L_{A}+L_{C})+\alpha_{B}L_{B}}+\beta_{B}\frac{\alpha_{B}L_{B}}{(1-\alpha_{B})(L_{A}+L_{C})+\alpha_{B}L_{B}}+\beta_{C}\frac{(1-\alpha_{B})L_{C}}{(1-\alpha_{B})(L_{A}+L_{C})+\alpha_{B}L_{B}}) \\ \times(\frac{\delta(1-\alpha_{B})L_{A}}{(1-\alpha_{B})(L_{A}+L_{C})+\alpha_{B}L_{B}}+\frac{\alpha L_{B}+(1-\alpha_{B})L_{C}}{(1-\alpha_{B})(L_{A}+L_{C})+\alpha_{B}L_{B}})L_{B}-\gamma L_{B}\text{ (Eq.2)} \end{matrix}$$

$$\begin{matrix} \frac{dL_{C}}{dt}=(\beta_{A}\frac{(1-\alpha_{C})L_{A}}{(1-\alpha_{C})(L_{A}+L_{B})+\alpha_{C}L_{C}}+\beta_{B}\frac{(1-\alpha_{C})L_{B}}{(1-\alpha_{C})(L_{A}+L_{B})+\alpha_{C}L_{C}}+\beta_{C}\frac{\alpha_{C}L_{C}}{(1-\alpha_{C})(L_{A}+L_{B})+\alpha_{C}L_{C}}) \\ \times(\frac{\delta(1-\alpha_{C})L_{A}}{(1-\alpha_{C})(L_{A}+L_{B})+\alpha_{C}L_{C}}+\frac{(1-\alpha_{C})L_{B}+\alpha_{C}L_{C}}{(1-\alpha_{C})(L_{A}+L_{B})+\alpha_{C}L_{C}})L_{C}-\gamma L_{C}\text{ (Eq.3)} \end{matrix}$$

In a homogenous system that all the quasispecies mixed evenly, dynamics of the different mutants and wild-type virus should keep a good synchronousness because the overall replication rate is the same for all the quasispecies. However, the synchronousness is not true for observed dynamics of HBV quasispecies during antiviral therapy. Drug-sensitive quasispecies are often gradually removed from patients and drug-resistant mutants may eventually predominant viral population in some of patients. Therefore, the system should not be homogenous for modeling dynamics of viral amounts. We introduced the relative affinity to the dynamic model to accommodate the heterogeneity of quasispecies. It was assumed that amount of the mutants x ($L_{x}^{local}$) in a single cell has quantitative relationship $L_{x}^{local}/L_{others}^{local}=(\alpha L_{x}^{globle})/[(1-\alpha)L_{others}^{globe}]$with overall amount of the mutants x ($L_{x}^{globle}$) in the system, where $L_{others}^{local}$indicates the amount of other quasispecies in a single cell and $L_{others}^{globe}$notes the total amount of other quasispecies in the system. The relative affinity has a range$1>a\geq0$.

The aforementioned dynamic model can be simplified for analysis of steady state of a viral population. For the viral population in a local area with either specified mutants, such as rtA181T or rtM204I mutants (indicated by U), and other mixed HBVs (indicated by M), dynamics of viral amount could be modeled as below where quasispecies were mixed well with each other (α = 0.5):

$$\frac{dL}{dt}=[\beta_{U}P_{U}+\beta_{M}(1-P_{U})][\delta P_{U}+(1-P_{U})]L-\gamma L\text{ (Eq.4)}$$

Changing rates of the viral amount ($\frac{dL}{dt}$) are determined by the difference of viral replication and clearance in a moment in time. The viral replication that was regulated by activities of polymerase (βU and βM, βU > βM), frequency of the specified mutants in population (PU), relative package efficiency of S protein of the specified mutants (δ), and the viral load at the moment (L). The viral clearance is ascertained by viral amount at the moment and the clearance efficiency (γ). In HBV infection, the PU evolves automatically to achieve a maximum of viral production rate during progress of infection because the proportion leading to the highest production rate takes significant evolutionary advantage. The Eq.4 has derivative

$\frac{\partial^{2}L}{\partial t\partial P_{U}}=L[2P_{U}(1-\delta)(\beta_{M}-\beta_{U})-(2-\alpha)\beta_{M}+\beta_{U}]\text{ (Eq.5)}$.

The derivative (Eq.5) is equal to zero when the increase rate of total viral load has its maximum. Therefore, analysis to the Eq. 6 revealed the feature of steady state of the viral population. Only when rtA181T/sW172* mutants and other mixed drug-sensitive quasispecies exist, can the frequency of rtA181T/sW172* mutants reach a steady state at$P_{U}=[(2-\delta)\beta_{M}-\beta_{U}]/[2(\beta_{M}-\beta_{U})(1-\delta)]\text{ (Eq.6)}$ with$1\geq P_{U}\geq0$, if the relative package efficiency of the truncated S protein of rtA181T/sW172* mutants is low ($\delta\leq0.5$). In contrast, when rtM204I mutants and other mixed quasispecies co-exist in the viral population, the frequency of rtM204I mutants could reach a steady state only at PU =1, if relative package efficiency of S protein of the rtM204I mutants is the similar as that of other HBVs (δ ≈ 1). In other words, in the antiviral therapy, frequency of the rtM204I mutants will eventually approximate to 1 in the viral population with existence of the rtM204I mutants.

**Supplementary text S2: The model with homogenous infection state**

In a single cell infected by two quasispecies A and B, the proportion of quasispecies in total are $P_{A}$and $P_{B}=1-P_{A}$, respectively. We give both activities of polymerases ($\beta_{A}$and $\beta_{B}$) and package efficiencies of S proteins ($\delta_{A}$ and $\delta_{B}$ ) are different from each other for the two different quasispecies. Overall activity of polymerases is therefore determined by weighted sum of two polymerase activities, i.e. $\beta_{A}P_{A}+\beta_{B}(1-P_{A})$. And overall package efficiency is given as $\delta_{A}P_{A}+\delta_{B}(1-P_{A})$. We assume virion production of the single cell ($R_{i}$) is determined by production of overall polymerase activity and package efficiency,

$$\frac{dR_{i}}{dt}=[\beta_{A}P_{A,i}+\beta_{B}(1-P_{A,i})][\delta_{A}P_{A,i}+\delta_{B}(1-P_{A,i})]$$

$$(\text{Eq.S1})$$

For a homogeneous viral group in size $S$ where all cells carried the two quasispecies in the same proportion, the overall virion production is given as$\frac{dR}{dt}=[\beta_{A}P_{A}+\beta_{B}(1-P_{A})][\delta_{A}P_{A}+\delta_{B}(1-P_{A})]S$. While the production of the single cells is a constant, i.e. $[\beta_{A}P_{A}+\beta_{B}(1-P_{A})][\delta_{A}P_{A}+\delta_{B}(1-P_{A})]\in C$, the overall virion production could be represented as $\frac{dR}{dt}=[\beta_{A}P_{A}+\beta_{B}(1-P_{A})][\delta_{A}P_{A}+\delta_{B}(1-P_{A})]R$ . Since the viral load was determined by both virion production and clearance, the viral load contributed by the viral group j could be represented as $\frac{dL_{j}}{dt}=[\beta_{A}P_{A}+\beta_{B}(1-P_{A})][\delta P_{A}+(1-P_{A})]L_{j}-\gamma L_{j}$. Where, $\tau$ is the clearance efficiency and $\delta=\delta_{A}/\delta_{B},\delta_{B}=1$. The above paragraph addresses the details for Eq. 4 of main text.

The above demonstration also gave the viral load of quasispecies A and B as

$$\begin{matrix} \frac{dL_{A,j}}{dt}=[\beta_{A}P_{A}+\beta_{B}(1-P_{A})][\delta P_{A}+(1-P_{A})]L_{A,j}-\gamma L_{A,j} \\ \frac{dL_{B,j}}{dt}=[\beta_{A}P_{A}+\beta_{B}(1-P_{A})][\delta P_{A}+(1-P_{A})]L_{B,j}-\gamma L_{B,j} \end{matrix}$$

**Supplementary text S3: The model with heterogeneous infection states**

Given$P_{A,i}=\frac{\alpha_{i}P_{A}}{\alpha_{i}P_{A}+(1-\alpha)_{i}(1-P_{A})}$ , we can rewrite the Eq. S1 as below,

$$\frac{dR_{i}}{dt}=[\beta_{A}\frac{\alpha_{i}P_{A}}{\alpha_{i}P_{A}+(1-\alpha_{i})(1-P_{A})}+\beta_{B}\frac{(1-\alpha_{i})P_{A}}{\alpha_{i}P_{A}+(1-\alpha_{i})(1-P_{A})}][\delta_{A}\frac{\alpha_{i}P_{A}}{\alpha_{i}P_{A}+(1-\alpha_{i})(1-P_{A})}+\delta_{B}\frac{(1-\alpha_{i})(1-P_{A})}{\alpha_{i}P_{A}+(1-\alpha_{i})(1-P_{A})}]$$

$$(\text{Eq.S2})$$

Follow the same manner of aforementioned group of homogeneous cells, the viral load of heterogeneous groups can be represented as

$\frac{dL_{A}}{dt}=\sum_{j} \{[\beta_{A}\frac{\alpha_{j}P_{A}}{\alpha_{j}P_{A}+(1-\alpha_{j})P_{A}}+\beta_{B}\frac{(1-\alpha_{j})P_{A}}{\alpha_{j}P_{A}+(1-\alpha_{j})P_{A}}][\delta_{A}\frac{\alpha_{j}P_{A}}{\alpha_{j}P_{A}+(1-\alpha_{j})P_{A}}+\delta_{B}\frac{(1-\alpha_{j})P_{A}}{\alpha_{j}P_{A}+(1-\alpha)_{j}P_{A}}]L_{A,j}-\gamma L_{A,j}\}$ .

For simplicity, we assume $\alpha=\alpha_{i},i\in\{1,2,...,n\}$ . The change of viral load of quasispecies A can be rewritten as

$\frac{dL_{A}}{dt}=[\beta_{A}\frac{\alpha P_{A}}{\alpha P_{A}+(1-\alpha)P_{A}}+\beta_{B}\frac{(1-\alpha)P_{A}}{\alpha P_{A}+(1-\alpha)P_{A}}][\delta_{A}\frac{\alpha P_{A}}{\alpha P_{A}+(1-\alpha)P_{A}}+\delta_{B}\frac{(1-\alpha)P_{A}}{\alpha P_{A}+(1-\alpha)P_{A}}]L_{A}-\gamma L_{A}$.

In the same manner, for a system with three quasispecies, we can present dynamics of the three quasispecies using the system of ordinary differential equations in Eq. 1-3 of main text.

**Supplementary text S4: Computer simulation.**

Observed fluctuation of rtA181T and rtM204I mutant frequencies in PVR and VB groups enabled us to assign parameters for the aforementioned dynamic model and further explore mechanism of HBV dynamics in the antiviral therapy.

In some cases, rtA181T mutants are dominant quasispecies of the viral population after a long-time treatment.[1] Therefore, the relative package efficiency of S protein of the rt181T mutants cannot be less than 0.5, because we have $P_{U}=[(2-\delta)\beta_{M}-\beta_{U}]/[2(\beta_{M}-\beta_{U})(1-\delta)]$and$P_{U}=1\Rightarrow\delta\geq0.5$ while$\beta_{U}>\beta_{M}\text{ and }\beta_{U}\to\infty$. However, the frequency of rtA181T mutants never exceeds 0.131 in our study. The rtA181T frequency observed in PVR group suggested that the relative package efficiency was likely to be no more than 0.5 ($P_{U}=[(2-\delta)\beta_{M}-\beta_{U}]/[2(\beta_{M}-\beta_{U})(1-\delta)]$ and$1\geq P_{U}\geq0\Rightarrow\delta\leq0.5$, while$\beta_{U}>\beta_{M}\text{ and }\beta_{U}<\infty$). Thus, we gave the relative package efficiency δ = 0.5 in our simulations.

As observed in our data, the viral load and frequencies of rtA181T mutant reached a relative stable state at week 12 of treatment in PVR group. We assume that the viral population would be in the stable state only when the frequency of rt181 mutants was around 0.1. Therefore, when activity of polymerase of drug-sensitive quasispecies is given as βM = 1 unit, we estimated activity of polymerase with rtA181T (βrtA181T) to be βrtA181T = 1.56 units because of the Eq. 6 ($0.1=(1.5-\beta_{rtA181T})/(1-\beta_{rtA181T})\text{ }\Rightarrow\text{ }\beta_{rtA181T}\text{=1.56}$units). Furthermore, polymerase activity of rtM204I mutants could be in a range from 1.04 to 1.56 units as the rtM204I mutants can eventually dominant a population with the presence of rtA181T mutants ($0\geq(1.5\times\beta_{rtM204I}-1.56)/(\beta_{rtM204I}-1.56)\text{ }\Rightarrow\text{ }\beta_{rtA181T}>\beta_{rtM204I}$ and$\text{1.56 >}\beta_{rtM204I}\geq1.04$). We assigned the polymerase activity of rtM204I mutants βrtM204I = 1.04 in this study. And therefore, the clearance rate (γ) of HBV in vivo was γ ≈1.003 unit ($\text{(0.5}\times\text{1.56+0.5}\times\text{1)}\times(0.5\times0.5+0.5\times1\text{)-}\gamma\text{=0 }\Rightarrow\text{ }\gamma=1.0032$).

During the treatment, drug-resistant mutants have better fitness than the wild-type virus. Assuming the rtA181T mutants reach its maximum replication, we gave the relative affinity of polymerases of rtA181T mutants by the function αrtA181T = (0.1-0.1frtA181T) / (0.8frtA181T+0.1) in our simulation 1-3 (Figure 3) because of the equation 0.1/0.9= (α frtA181T)/ [(1-α)(1- frtA181T)]. While the viral population of rtA181T mutants and wild-type viruses can be stable during the treatment of PVR.7, we explicitly assume drug-sensitive viruses have relative affinity of polymerases (α) the same as that of rtA181T mutants because, in the steady state, both the rtA181T mutant and wild-type virus must have the same relative affinity α = 0.5 to achieve a maximum replication rate. To achieve a maximum replication of rtM204I mutants in VB.7 and VB.9, relative affinity of polymerases (α) of rtM204I mutants should be high (αrtM204I →1). In the scenarios of αrtM204I ≫ 0.99, frequency of rtA181T mutants will never be ≥0.1 in simulations which is in contradiction to our sequencing data (Table S2). We therefore empirically gave αrtM204I = 0.99 in the simulation 2 and 3 for simplicity.

Computer simulation using the Eq.1-3 was conducted in MATLAB with the aforementioned parameters and different initial viral amounts. In the simulation 1, we assumed only rtA181T mutants and drug-sensitive viruses presented in viral population and their viral amounts were 10^5^ and 10^8^, respectively; in the simulation 2, we assumed both rtA181T and rtM204I mutants and the drug-sensitive viruses presented in viral population and their viral amounts were 10^5^, 10^4^, and 10^8^, respectively; in the simulation 3, we assumed both rtA181T and rtM204I mutants and the drug-sensitive viruses presented in viral population and their viral amounts were 10^3^, 10^4^, and 10^8^, respectively.

**Supplementary text S5: Drug-resistant mutations occurred before antiviral treatment.**

We denote mutation rate per HBV sequence in a moment in time as μ. In a moment in time, mutation events (*X*) happened in a viral population is in proportion to the amount of viruses$X=\mu L$, where *L* is the size of viral population at the moment. When μ is a constant, the total number of mutation events is solely determined by the amount of viruses. In a period from time t to t+1, we have the total number of mutation events $X=\sum_{i=t}^{t+1} uL_{i}$.

We assume the dynamics of total amount of viruses is given as$\frac{dL}{dt}=kL$, where k is production rate of viral population. When amount of viruses at time 0 is assigned as *L^0^*, the amount of viruses at the time t (noted by *L^t^*) can be obtained in$L^{t}=L^{0}e^{kt}$. Therefore, total mutation events (*X*) during time t_1_ to t_2_ can be achieved in the equation$X=\int_{t1}^{t2} \mu L^{0}e^{kt}dt=\frac{\mu L^{0}}{k}(e^{kt_{2}}-e^{kt_{1}})$.

In PVR group, we have viral loads around 10^9^, 10^5^, or 10^4^ at the baseline, week 12, or week 24 and thereafter, respectively. Therefore, we give *k^w0~12^ = -0.768, k^w12~24^ = -0.192* and *k^w24~w52^ = 0* for periods during week 0 to week 12, week 12 to week 24, and week 24 to week 52 respectively. Our analysis shows rate of accumulation of mutation events decreases rapidly after the beginning of the treatment. For example, the number of mutation events occurred in the 6^th^ week is only 1.50% of that of the 1^st^ week based on the aforementioned formula. Actually, mutation events occurred in the one-year treatment (52 weeks) is only about 32.5% of that happened in one month immediately before the treatment. Because the patients usually had high viral levels over a long period (far more than one month) before the antiviral therapy, drug-resistant mutations are more likely to exist before the beginning of antiviral therapy.

**Figure S1. Phylogenies of HBVs from 10 patients with typical virological breakthrough.**

The lineages carrying rt181T and rt204I were marked in blue and red, respectively. Obviously, these two mutants were derived from independent phylogenetic origins. The time points that lineages were observed were shown on different rims of the circles in different colors (week 0, sky blue; week12, spring green ; week 24, yellow green ; week 36, orange; week 52, dark red). A significant decrease of pairwise difference of the subject's viral sequences was observed at week 52 (after virologcial breakthrough) compared to week 36 (before virological breakthrough).

**Figure S2. The distribution of sequence read length.**

The read length of most sequences converges at approximately 380bp, matching exactly the length of PCR products we sequenced.

**Figure S3. Mismatch error rate of the plasmid.**

The plasmid was sequenced repeatedly in 6 separate runs of 454 GS FLX^+^ System and no more than 3 nucleotides per run had a mismatch error rate ≥1%.


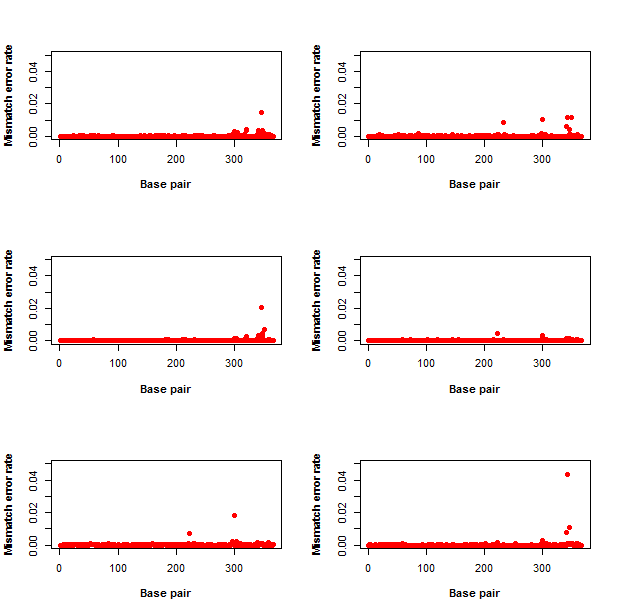


**Table S1. HBV DNA levels (log^10^ copies/ml) and HBsAg levels (log^10^ IU/ml). HBV DNA levels were determined using the Roche COBAS Taqman. HBsAg were assayed using Architect I2000 (Abbott Laboratories). *Patients VB.1, VB.2 and VB.3 experienced VB at week 36 of treatment, and their serum samples at week 52 was not involved in this study because LdT monotherapy was ended after VB.**

| **Patient/**  **Time** | **HBV DNA levels** | | | | | **HBsAg levels** | |
| --- | --- | --- | --- | --- | --- | --- | --- |
|  | **W0**  **(%)** | **W 12**  **(%)** | **W 24**  **(%)** | **W 36**  **(%)** | **W 52**  **(%)** | **W0**  **(%)** | **W 24**  **(%)** |
| **CVR.1** | 8.479 | 4.333 |  |  |  | 4.333 | 2.954 |
| **CVR.2** | 9.316 | 4.977 |  |  |  | 4.977 | 3.634 |
| **CVR.3** | 8.321 | 3.638 |  |  |  | 3.638 | 3.216 |
| **CVR.4** | 8.806 | 4.884 |  |  |  | 4.884 | 2.448 |
| **CVR.5** | 9.091 | 4.256 |  |  |  | 4.256 | 3.799 |
| **CVR.6** | 9.091 | 4.558 |  |  |  | 4.558 | 4.109 |
| **CVR.7** | 9.730 | 4.696 |  |  |  | 4.696 | 3.188 |
| **CVR.8** | 8.499 | 4.170 |  |  |  | 4.170 | 3.281 |
| **CVR.9** | 9.539 | 4.665 |  |  |  | 4.665 | 3.185 |
| **CVR.10** | 7.681 | 3.185 |  |  |  | 3.185 | 3.522 |
| **PVR.1** | 8.519 | 4.739 | 4.068 | 3.593 |  | 3.350 | 3.275 |
| **PVR.2** | 9.360 | 5.730 | 4.947 | 4.920 |  | 4.704 | 4.376 |
| **PVR.3** | 7.990 | 5.081 | 4.528 | 3.750 |  | 4.014 | 3.158 |
| **PVR.4** | 9.212 | 5.528 | 4.952 | 4.761 |  | 4.639 | 4.654 |
| **PVR.5** | 9.532 | 5.663 | 4.840 | 4.471 |  | 4.532 | 3.397 |
| **PVR.6** | 8.611 | 4.134 | 3.159 | 3.917 |  | 4.105 | 4.224 |
| **PVR.7** | 9.738 | 5.794 | 5.349 | 4.998 |  | 4.539 | 3.766 |
| **PVR.8** | 9.782 | 5.666 | 5.138 | 4.615 |  | 4.713 | 3.355 |
| **PVR.9** | 9.311 | 5.242 | 5.025 | 4.550 |  | 4.171 | 3.303 |
| **PVR.10** | 9.072 | 5.207 | 5.221 | 5.203 |  | 4.740 | 4.155 |
| **VB.1*** | 8.516 | 3.844 | 4.332 | 8.786 |  | 4.652 | 4.255 |
| **VB.2*** | 8.689 | 5.185 | 4.310 | 5.855 |  | 4.197 | 4.243 |
| **VB.3*** | 9.672 | 6.235 | 3.258 | 5.730 |  | 5.442 | 3.087 |
| **VB.4** | 9.757 | 5.591 | 5.115 | 4.151 | 5.348 | 4.696 | 3.861 |
| **VB.5** | 10.154 | 6.191 | 5.552 | 4.523 | 6.630 | 5.246 | 3.995 |
| **VB.6** | 10.417 | 5.697 | 3.037 | 3.455 | 7.140 | 5.458 | 4.151 |
| **VB.7** | 9.377 | 5.301 | 4.221 | 3.115 | 6.298 | 4.697 | 3.858 |
| **VB.8** | 8.430 | 5.130 | 3.741 | 3.366 | 4.712 | 3.723 | 3.710 |
| **VB.9** | 8.309 | 4.872 | 3.352 | 4.003 | 6.944 | 3.709 | 3.856 |
| **VB.10** | 9.010 | 6.151 | 5.154 | 5.512 | 8.724 | 4.780 | 3.400 |

**Table S2.** Frequencies of rtA181T and rtM204I mutants in 3 groups.

| **Patient/**  **Time** | **rtA181T mutations** | | | | | **rtM204I mutations** | | | | |
| --- | --- | --- | --- | --- | --- | --- | --- | --- | --- | --- |
|  | **W0**  **(%)** | **W 12**  **(%)** | **W 24**  **(%)** | **W 36**  **(%)** | **W 52**  **(%)** | **W0**  **(%)** | **W 12**  **(%)** | **W 24**  **(%)** | **W 36**  **(%)** | **W 52**  **(%)** |
| **CVR.1** | 0.00 | 0.00 | / | / | / | 0.00 | 0.00 | / | / | / |
| **CVR.2** | 0.04 | 0.02 | / | / | / | 0.00 | 0.03 | / | / | / |
| **CVR.3** | 0.24 | 0.00 | / | / | / | 0.16 | 0.00 | / | / | / |
| **CVR.4** | 0.06 | 0.14 | / | / | / | 0.00 | 0.03 | / | / | / |
| **CVR.5** | 0.04 | 0.00 | / | / | / | 0.04 | 0.03 | / | / | / |
| **CVR.6** | 0.15 | 0.02 | / | / | / | 0.00 | 0.00 | / | / | / |
| **CVR.7** | 0.03 | 0.86 | / | / | / | 0.00 | 0.00 | / | / | / |
| **CVR.8** | 0.03 | 0.00 | / | / | / | 0.03 | 0.00 | / | / | / |
| **CVR.9** | 0.01 | 0.00 | / | / | / | 0.00 | 0.00 | / | / | / |
| **CVR.10** | 0.05 | 0.03 | / | / | / | 0.05 | 0.00 | / | / | / |
| **PVR.1** | 0.51 | 7.98 | 3.47 | 2.04 | / | 0.06 | 0.02 | 0.02 | 0.00 | / |
| **PVR.2** | 0.38 | 1.66 | 5.41 | 10.59 | / | 0.03 | 0.02 | 0.23 | 0.53 | / |
| **PVR.3** | 0.15 | 3.50 | 3.71 | 2.91 | / | 0.00 | 0.27 | 0.02 | 0.03 | / |
| **PVR.4** | 0.00 | 0.17 | 0.00 | 0.64 | / | 0.00 | 0.35 | 0.03 | 0.97 | / |
| **PVR.5** | 0.21 | 3.52 | 5.10 | 7.44 | / | 0.06 | 0.02 | 0.22 | 0.88 | / |
| **PVR.6** | 0.12 | 2.99 | 7.06 | 3.48 | / | 0.00 | 0.10 | 0.00 | 0.04 | / |
| **PVR.7** | 0.27 | 4.19 | 6.05 | 6.02 | / | 0.00 | 0.04 | 0.43 | 0.11 | / |
| **PVR.8** | 0.15 | 3.01 | 4.34 | 6.05 | / | 0.00 | 0.04 | 0.41 | 0.00 | / |
| **PVR.9** | 0.23 | 6.94 | 4.49 | 9.74 | / | 0.00 | 0.36 | 0.23 | 0.29 | / |
| **PVR.10** | 0.00 | 0.14 | 0.11 | 0.31 | / | 0.05 | 0.24 | 0.22 | 0.00 | / |
| **VB.1** | 0.05 | 0.00 | 0.02 | 0.08 | / | 0.05 | 0.14 | 99.52 | 99.40 | / |
| **VB.2** | 0.00 | 0.00 | 0.00 | 0.03 | / | 0.00 | 0.12 | 35.22 | 97.75 | / |
| **VB.3** | 0.00 | 0.52 | 1.26 | 1.20 | / | 0.00 | 0.30 | 0.11 | 0.31 | / |
| **VB.4** | 0.38 | 8.11 | 13.11 | 12.19 | 3.99 | 0.00 | 0.14 | 0.07 | 0.60 | 69.01 |
| **VB.5** | 0.03 | 0.49 | 0.37 | 2.80 | 0.02 | 0.00 | 0.03 | 0.27 | 0.43 | 99.05 |
| **VB.6** | 0.11 | 2.56 | 2.12 | 0.03 | 0.02 | 0.00 | 0.09 | 0.41 | 44.60 | 99.62 |
| **VB.7** | 0.64 | 11.15 | 12.43 | 11.96 | 0.02 | 0.08 | 0.00 | 0.08 | 0.01 | 99.64 |
| **VB.8** | 0.13 | 0.68 | 2.18 | 0.03 | 0.00 | 0.07 | 0.00 | 0.06 | 2.80 | 97.22 |
| **VB.9** | 0.02 | 0.52 | 0.00 | 0.05 | 0.83 | 0.05 | 0.19 | 0.00 | 1.09 | 94.06 |
| **VB.10** | 0.20 | 1.63 | 5.06 | 5.02 | 0.00 | 0.00 | 0.14 | 0.19 | 30.00 | 99.38 |

W0: baseline, W12: week 12, W24: week 24, W36: week 36, W52: week 52.

**Table S3. Types and frequencies of sW172 and sW196 mutations in 3 groups.**

| **Patient/**  **Time** | **sW172 mutations** | | | | | **sW196 mutations** | | | | |
| --- | --- | --- | --- | --- | --- | --- | --- | --- | --- | --- |
|  | **W0**  **(%)** | **W 12**  **(%)** | **W 24**  **(%)** | **W 36**  **(%)** | **W 52**  **(%)** | **W0**  **(%)** | **W 12**  **(%)** | **W 24**  **(%)** | **W 36**  **(%)** | **W 52**  **(%)** |
| **CVR.1** | **0.00** | **0.00** | **/** | **/** | **/** | **0.00** | **0.00** | **/** | **/** | **/** |
| **CVR.2** | ***0.04** | ***0.02** | **/** | **/** | **/** | **.050000000000000000000000000000000000000000000000000000000000000000000000000000000000000000000000000000000000000000000000000000.00** | ***0.03** | **/** | **/** | **/** |
| **CVR.3** | ***0.15**  **C0.10** | **0.00** | **/** | **/** | **/** | ***0.16** | **0.00** | **/** | **/** | **/** |
| **CVR.4** | ***0.03**  **C0.03** | ***0.14** | **/** | **/** | **/** | **0.00** | **L0.03** | **/** | **/** | **/** |
| **CVR.5** | ***0.04** | **0.00** | **/** | **/** | **/** | ***0.04** | **L0.03** | **/** | **/** | **/** |
| **CVR.6** | ***0.09**  **C0.06** | ***0.02** | **/** | **/** | **/** | **0.00** | **0.00** | **/** | **/** | **/** |
| **CVR.7** | **C0.03** | **0.00** | **/** | **/** | **/** | **0.00** | **0.00** | **/** | **/** | **/** |
| **CVR.8** | **C0.03** | **0.00** | **/** | **/** | **/** | **L0.03** | **0.00** | **/** | **/** | **/** |
| **CVR.9** | ***0.01** | **0.00** | **/** | **/** | **/** | **0.00** | **0.00** | **/** | **/** | **/** |
| **CVR.10** | **C0.05** | ***0.03** | **/** | **/** | **/** | ***0.05** | **0.00** | **/** | **/** | **/** |
| **PVR.1** | ***0.28**  **C0.24** | ***7.27**  **C0.71** | ***3.47** | ***2.04** | **/** | ***0.02**  **L0.02** | **L0.03** | ***0.02** | ***0.09**  **L0.03** | **/** |
| **PVR.2** | ***0.30**  **C0.08** | ***1.65**  **C0.01** | ***5.41** | ***9.82**  **C0.77** | **/** | ***0.03** | ***0.02** | ***0.23** | ***0.19**  **L0.34** | **/** |
| **PVR.3** | ***0.15** | ***3.5** | ***3.71** | ***2.91** | **/** | ***0.02** | ***0.24**  **L0.02** | ***0.02** | ***0.03** | **/** |
| **PVR.4** | **0.00** | ***0.15**  **C0.02** | **0.00** | ***0.87** | **/** | **0.00** | ***0.35** | ***0.03** | ***0.13**  **L 0.84** | **/** |
| **PVR.5** | ***0.17**  **C0.04** | ***3.51**  **C0.01** | ***5.10** | ***7.44** | **/** | ***0.06** | ***0.02** | ***0.22** | ***0.84**  **L0.03** | **/** |
| **PVR.6** | ***0.08**  **C0.04** | ***2.99** | ***7.06** | ***3.48** | **/** | **0.00** | ***0.10** | **0.00** | ***0.04** | **/** |
| **PVR.7** | ***0.23**  **C0.04** | ***4.18**  **C0.01** | ***6.05** | ***5.85**  **C0.16** | **/** | ***0.03** | ***0.04** | ***0.43** | ***0.04**  **L0.07** | **/** |
| **PVR.8** | ***0.11**  **C0.04** | ***3.00**  **C0.01** | ***4.34** | ***5.49**  **C0.56** | **/** | **0.00** | ***0.04** | ***0.06**  **L0.35** | **0.00** | **/** |
| **PVR.9** | ***0.23** | ***6.91**  **C0.03** | ***4.49** | ***9.19**  **C0.54** | **/** | **0.00** | ***0.32**  **L0.03** | ***0.17**  **L0.02** | ***0.29** | **/** |
| **PVR.10** | **0.00** | ***0.14** | ***0.09**  **C0.02** | ***0.31** | **/** | **L0.05** | ***0.24** | ***0.22** | **0.00** | **/** |
| **VB.1** | ***0.05** | **0.00** | ***0.02** | ***0.08** | **/** | **L0.05** | **L0.14** | **L99.52** | **L99.35**  ***0.05** | / |
| **VB.2** | **0.00** | **0.00** | **0.00** | ***0.03** | **/** | **0.00** | **L0.12** | **L35.22** | **L96.90**  **S0.84** | **/** |
| **VB.3** | **0.00** | ***0.52** | ***0.99**  **C0.27** | ***1.11**  **C0.08** | **/** | **0.00** | ***0.20**  **L0.08**  **S0.01** | ***0.11** | ***0.03**  **L0.27** | **/** |
| **VB.4** | ***0.38** | ***8.11** | ***13.11** | ***12.19** | ***3.99** | **0.00** | ***0.12**  **L0.02** | ***0.02**  **L0.02**  **S0.02** | ***0.03**  **L0.58** | ***0.14**  **L68.87** |
| **VB.5** | **C0.03** | ***0.49** | ***0.35**  **C0.02** | ***2.80** | **C0.02** | ***0.01** | ***0.01**  **L0.02** | ***0.03**  **L0.25** | ***0.03**  **L0.19**  **S0.22** | **L88.11**  **S10.94** |
| **VB.6** | ***0.11** | ***2.50**  **C0.06** | ***2.01**  **C0.11** | ***0.03** | ***0.02** | **0.00** | ***0.09** | ***0.35**  **S0.06** | ***L44.60** | **L99.62** |
| **VB.7** | ***0.94** | ***11.15** | ***12.43** | ***11.96** | **C0.02** | ***0.08** | **0.00** | ***0.05**  **L0.03** | ***0.01** | **L99.64** |
| **VB.8** | ***0.10**  **C0.03** | ***0.68** | ***2.18** | ***0.03** | **0.00** | ***0.07** | **0.00** | ***0.06** | **L2.80** | **L92.79**  **S 4.41** |
| **VB.9** | ***0.02** | ***0.52** | **0.00** | ***0.05** | ***0.83** | ***0.05** | ***0.19** | **0.00** | ***0.04**  **L1.80** | **L 93.64**  **S 0.66** |
| **VB.10** | ***0.14 C0.07** | ***1.63** | ***4.81**  **C0.24** | ***5.02** | **0.00** | **0.00** | ***0.14** | ***0.12**  **L0.08** | ***0.03**  **L29.88**  **S0.09** | **L 99.29**  **S0.09** |

W0: baseline, W12: week 12, W24: week 24, W36: week 36, W52: week 52.

W=Tryptophan, *=stop codon, C=Cysteine, L=leucine, S=serine.

**References:**

1 Yatsuji H, Noguchi C, Hiraga N*, et al.*.Emergence of a novel lamivudine-resistant hepatitis B virus variant with a substitution outside the YMDD motif.*Antimicrob Agents Chemother* 2006;**50**:3867-74.
